# Preventing the onset of diabetes with precision delivery of mesenchymal stem cells to the pancreas in a preclinical model

**DOI:** 10.1186/s13287-025-04807-3

**Published:** 2025-11-23

**Authors:** Reza Yarani, Rosita Primavera, Shashank Chetty, Jing Wang, Simranjeet Kaur, Martin Haupt-Jorgensen, Flemming Pociot, Avnesh S. Thakor

**Affiliations:** 1 Department of Radiology, Interventional Radiology Innovation at Stanford (IRIS), Stanford University, Palo Alto, CA 94304 USA; 2 Translational Type 1 Diabetes Research, Department of Clinical Research, Steno Diabetes Center, 2730 Copenhagen, Herlev Denmark; 3 Department of Pathology, Bartholin Institute, Rigshospitalet, Copenhagen University, 2200 Copenhagen, Denmark; 4 Faculty of Health and Medical Sciences,, Institute for Clinical Medicine, University of Copenhagen, 2200 Copenhagen, Denmark

**Keywords:** Mesenchymal stem cells, Precision delivery, Intra-arterial delivery, Glycemic regulation, Type 1 diabetes

## Abstract

**Background:**

Mesenchymal stem cells (MSCs) represent a promising regenerative therapy associated with anti-inflammatory, anti-apoptotic, and pro-proliferative properties. Despite their significant potential for treating diabetes, the clinical application of MSCs has been hindered mainly due to the low number of intravenously (IV) injected cells that successfully reach the pancreas.

**Methods:**

In this study, we assessed the protective potential of adipose tissue-derived MSCs (AD-MSCs) in preventing the development of diabetes using a streptozotocin (STZ)-induced diabetic rat model. AD-MSCs were administered 4 h after STZ either via conventional IV injection or through direct intra-arterial (IA) administration into the pancreas. Healthy (no STZ, no MSCs) and diabetic (STZ, no MSCs) control groups were also included.

**Results:**

Our proof-of-concept data indicate that IA administration of AD-MSCs allowed rats to maintain glycemic control and respond appropriately to glucose challenges following STZ treatment, comparable to healthy controls. In contrast, rats receiving IV AD-MSCs developed hyperglycemia and failed to respond adequately to glucose challenges. In vitro studies demonstrated that AD-MSCs can enhance the viability and function (i.e. insulin secretion) of injured islets.

**Conclusion:**

Local IA delivery of AD-MSCs into the pancreatic circulation effectively prevents the onset of diabetes in a preclinical rat model, highlighting the need for considering delivery techniques to ensure MSCs can reach their targets in vivo.

**Supplementary Information:**

The online version contains supplementary material available at 10.1186/s13287-025-04807-3.

## Background

Type 1 diabetes (T1D) is characterized by immune-mediated destruction of pancreatic β cells, leading to insulin deficiency and hyperglycemia, necessitating lifelong exogenous insulin therapy. Hence, given the globally rising incidence of this disease, developing therapeutic approaches to protect β cells and maintain in vivo glycemic control is of great importance.

One promising approach is the use of mesenchymal stem cells (MSCs), which are heterogeneous, multipotent cells capable of self-renewal. MSCs have demonstrated efficacy in treating different diseases [1, 2], including diabetes [3–6], given their anti-inflammatory, immunomodulatory, pro-angiogenic, and anti-fibrotic properties. Indeed, studies have shown that MSCs can help protect pancreatic islets in vitro and in vivo when used in islet transplantation [3]. However, the ability of MSCs to protect and regenerate pancreatic islets in diabetes has been limited, primarily due to their inability in reaching the pancreas following conventional intravenous (IV) injection, given that the majority of cells end up in the lung and/or reticuloendothelial system [7, 8]. Indeed, clinical trials have reported only modest improvement in metabolic control in diabetic patients treated with IV MSCs [4, 5]. To maximize the therapeutic potential of MSCs, it is crucial to deliver them precisely to the target site, in this case, the pancreas. To address this, we developed a technique to deliver therapeutics directly to the pancreas via locoregional intra-arterial (IA) injection [9, 10] in small animal models, in which the vascular supply of the pancreas is carefully isolated using microsurgical techniques. Such techniques are clinically translatable using minimally invasive, endovascular, image-guided approaches.

In the present study, we developed a diabetic animal model that mimics the gradual onset of hyperglycemia observed in stages 1–2 of type 1 diabetes, characterized by progressive loss of β cell function [11]. Streptozotocin (STZ), which enters β cells via the glucose transporter type 2 (GLUT2) receptor, causes β cell injury [12] through the generation of reactive oxygen species (ROS) and inflammation [13, 14]. Over time, this injury results in the dysfunction and eventual death of β cells, causing hyperglycemia [15, 16], with the degree of β cell loss dependent on the dose of STZ administered. In a optimized model, characterized by a gradual loss of β cells, we assessed the ability of adipose tissue-derived MSCs (AD-MSCs) to protect islets from STZ-induced injury when administered by either IV or IA injection. AD-MSCs were chosen as they possess promising regenerative [17, 18], immunomodulatory [19, 20], and antiapoptotic [21–23] properties and can be obtained abundantly with a high yield from biological material acquired by liposuction [24]. Our findings demonstrate that AD-MSCs can protect injured islets from cell death and enhance their viability. Importantly, this effect in vivo was observed mainly when cells were administered directly into the pancreas via IA injection and not following IV injection.

## Methods

### Reagents

Histopaque (R)-1077 [10771], histopaque (R)-1119 [11191], heat-inactivated fetal bovine serum (FBS) [F0926], propidium iodide (PI) [P4170], fluorescein diacetate (FDA) [F7378], D-glucose [G7528], bovine serum albumin (BSA) [A2153], collagenase from clostridium histolyticum [C6885], accutase [A6964], and streptozotocin (STZ) [S0130] were purchased from Sigma Aldrich (Saint Louis, Missouri, USA). Roswell Park Memorial Institute (RPMI) medium, without phenol red [11835030], phosphate-buffered saline (PBS) [10010023], and penicillin/streptomycin [15140122], was obtained from GIBCO (Invitrogen Corporation, Waltham, Massachusetts, USA). Hank’s Balanced Salt Solution (HBSS) without calcium and magnesium, and low-glucose Dulbecco’s modified Eagle’s Minimal Essential Medium (DMEM) were purchased from Hyclone (Logan, Utah, USA). All other products and reagents were of analytical grade.

### Animals

All experiments in the present study were approved by the Administrative Panel on Laboratory Animal Care at Stanford University and were carried out per the Institutional Animal Care and Use Committee (IACUC) approval (31947). Rats were maintained in a pathogen-free environment at the animal facility in the Canary Center at Stanford University with free access to food and water with a 12 h:12 h light-dark cycle.

### Cells

Human AD-MSCs (CELLvo^™^ hAD-MSCs, catalog number: AD-100-000 and lot number: AD 18 − 002) used in this study were obtained from StemBioSys at passage number P1 (TX, USA). Cells were characterized using the minimal criteria defined by the International Society for Cell & Gene Therapy [25, 26]. AD-MSCs were cultured in low-glucose DMEM (supplemented with 1% (v/v) penicillin/streptomycin solution, 10% (v/v) FBS, and L-glutamine and incubated at 37˚C in a humidified atmosphere containing 5% CO_2_. Cells were passaged when they reached 80% confluency in a treated tissue culture plate (150 mm x 20 mm, CELLTREAT Scientific Products, Massachusetts, USA). AD-MSCs from passages 4–6 were harvested using accutase solution, pelleted by centrifugation at 300 x g for 5 min, mixed with trypan blue, and counted using a Countess automated cell counter (Life Technologies, USA). These cells were then used fresh for all studies.

### In vivo STZ dose-response study

Male and female Wistar rats, 8–10 weeks of age (Charles River Laboratories, USA), were used. STZ was freshly prepared using 0.1 M ice-cold citrate buffer (pH 4.5) and administered at different doses: 10, 20, 30, 40, 50, and 60 mg/kg by one intraperitoneal (IP) injection into the animals. For the lower concentrations of STZ (10, 20, and 30 mg/kg), we included *N* = 2 female and *N* = 2 male rats, as we did not anticipate any toxic effects at these doses. For the higher concentrations of STZ (40, 50, and 60 mg/kg), we used *N* = 4 female and *N* = 4 male rats to ensure reproducibility and to account for any refined differences between dosages. The initial body weights of the rats averaged 372.7 ± 31.8 g and 281.8 ± 29.0 g for males and females, respectively.

The following evaluations were then performed:

1. Non-fasting blood glucose (BG) and weight measurement: Non-fasting BG levels were measured daily via the tail vein using Contour blood glucose strips (Ascensia Diabetes Care, USA). Rats were considered diabetic when BG levels were ≥ 250 mg/dL over three consecutive measurements [27–29]. Weight measurements were done daily for all animals.
2. Intraperitoneal glucose tolerance test (IPGTT): IPGTTs were performed on day 14. Rats were fasted overnight for 16 h, and their BG levels were measured before and after an IP injection of glucose (dose: 2 g/kg) at 0, 30, 60, 90, and 120 min. The glucose solution was prepared by dissolving glucose in sterile saline at a concentration of 400 mg/mL. To ensure sterility, the solution was then filtered through a 0.22 μm filter. If not used immediately, it was stored in a sterile container at 4 °C and was to be used within 2 days to maintain stability.
3. Histological analysis: pancreatic tissue was harvested at euthanasia on day 14, fixed in 10% formalin, and embedded in paraffin. The tissue was then sectioned, cut into 5-µm-thick slices, and stained with Hematoxylin and Eosin (H&E) to facilitate assessment of overall islet morphology and damage. Stained sections were analyzed using a NanoZoomer slide scanner 2.0-RS (Hamamatsu Photonics, Japan) and NDP.VIEW2 software.

Rats were euthanized on day 14 through inhalation of carbon dioxide, in accordance with humane guidelines for the ethical treatment of laboratory animals.

### AD-MSCs effect in STZ-treated rats

Male Wistar rats received a single intraperitoneal injection of 50 mg/kg STZ, administered 4 h prior to the injection of AD-MSCs. We specifically selected a dose of 50 mg/kg STZ for this experiment because it was determined to be the optimal concentration that induces islet damage without completely destroying the islets, as demonstrated in our STZ dose-response study. A total of 20 rats were randomly allocated into four groups: Healthy (no STZ, no MSCs, *N* = 5), Diabetic (STZ, no MSCs, *N* = 5), IV (STZ, IV MSCs, *N* = 5), and IA (STZ, IA MSCs, *N* = 5). For the IA and IV groups, rats received 1.5 × 10^6^ AD-MSCs in 100 µL of sterile phosphate-buffered saline (PBS) 4 h after STZ injection, and BG-level measurements and IPGTT assessments were performed for all groups as explained above.

1. Intra-arterial (IA) delivery of AD-MSCs: All surgical procedures were performed in a sterile procedure room with sterile equipment. IA delivery was performed as previously described [9, 30]. In brief, rats were anesthetized (isoflurane 2%), and the abdominal wall was opened (2–3 cm midline incision). The celiac artery, distal splenic artery, and gastroduodenal artery were exposed and micro-dissected. The distal branches of the splenic artery, hepatic artery, left gastric artery, and duodenal branches of the distal gastroduodenal artery were temporarily ligated. The celiac artery was cannulated with a 36-gauge NANOFIL needle (World Precision Instruments, USA), ensuring that at least 5 mm of the needle was placed within the vessel. AD-MSCs were injected slowly, after which the needle was removed, and all temporary ligation sutures were released – this was done within 5 min of ligation to avoid any end-organ ischemia. Gentle pressure was applied to the celiac artery using a cotton tip to achieve hemostasis. Finally, the abdomen was closed, and the rats were individually recovered for 24 h following surgery. All animals were then monitored daily for 14 days.
2. Intra-venous (IV) delivery of AD-MSCs: All surgical procedures were performed in a sterile procedure room with sterile equipment. IV delivery was performed via a tail vein injection.
3. AD-MSC biodistribution: First, 2 × 10^6^ AD-MSCs/mL was mixed with 5µL of Vybrant^™^ Dil cell-labeling solution and incubated for 20 min at 37˚C. After incubation, the cells were centrifuged at 300 x g for 5 min, and the supernatant was removed. The cells were then gently washed three times in PBS to remove any excess dye. Cells were counted, and 1.5 × 10^6^ AD-MSCs were injected into rats via IA or IV injections. Rats were then sacrificed at days 1 and 5 post-injection, and the organs were harvested and imaged using a Lago Optical Imaging System [excitation:535 nm, emission:590 nm, exposure:200s, excitation power: 10%].

At the end of the study, rats were euthanized through inhalation of carbon dioxide, in accordance with humane guidelines for the ethical treatment of laboratory animals.

### In vitro STZ dose-response study

For the dose-response study, pancreatic islets were isolated and purified from male Wistar rats, as previously described [31], and then exposed to 0, 0.1, 0.2, 0.3, 0.5, 1, and 10 mM of STZ. Cell viability and death were evaluated after 48 h.

1. Islet isolation: Rats were anesthetized (2% isoflurane), the pancreas exposed, and the pancreatic duct isolated and cannulated. After placing a hemostat clamp on the bile duct near the opening of the small intestine, the pancreas was inflated by injecting 5 mL of cold collagenase (1 mg/ml in HBSS) through the bile duct. Following distension, the pancreas was carefully dissected free from its surrounding tissues and incubated for 10 min in a 37 °C water bath. The islets were purified using a 40-mesh sieve, then subjected to gradient centrifugation on Histopaque-1119 and 1077 at 1000 x g for 30 min at 4 °C. Islets were handpicked using a stereomicroscope (Nikon SMZ745, USA) and cultured in RPMI without phenol red with 10% FBS (v/v), 1% penicillin/streptomycin, and L-glutamine in an incubator with 5% CO_2_ at 37 °C.
2. Islet viability assessment: The islets were assessed using a fluorescence-based live/dead assay with FDA (green) and PI (red) to distinguish viable from dead cells. Fluorescent imaging was performed using confocal microscopy (Leica TSC SP8X White Laser Confocal Microscope), and the percentage of live islets was determined as follows:

$$\:Live\:islets\:\left(\%\right):\:\frac{green\:fluorescence}{red+green\:fluorescence}\:X\:100$$

The fluorescence intensity was quantified across the entire islet, and the evaluation was performed manually using ImageJ software.

3. Cell death assay: Islets were assessed using a cell death detection ELISA kit (cat no. 11774425001), following the manufacturer’s instructions (Sigma-Aldrich, Germany). Here, islets were seeded in 96-well plates (30 islets/per well). After 48 h, islets were lysed for 30 min in 300 µl lysis buffer, then centrifuged at 200 x g for 10 min. 20 µl supernatant per sample was mixed with 80 µl immunoreagent solution in each well. The plate was incubated for 2 h at 300 rpm. The solution was thoroughly removed by inversion and tapping onto absorbent paper. Each well was rinsed 3 times with 250 µl incubation buffer. 100 µl 2,2′-azino-bis(3-ethylbenzothiazoline-6-sulfonic acid) (ABTS) solution was added to each well on a plate shaker (250 rpm) for color development for 5–10 min. 100 µl/well stop solution was added, and the absorbance was measured at 405 nm and 490 nm (490 nm readings were subtracted from the 405 nm readings).

### AD-MSCs assessment with STZ-stressed Islets

Based on data from the dose-response study, experiments were performed using 0.2 mM STZ in a transwell system for 48 h in the absence and presence of AD-MSCs (MSCs were placed in the top chamber, and rat islets were placed in the bottom chamber). Here, only 2% FBS was used. After 48 h, the following assessments were performed.

1. Nitric Oxide (NO) measurement: Islets alone and with AD-MSCs were seeded in trans-well plates, with 20 islets in each well, and incubated with and without 0.2 mM STZ. After 48 h, the supernatant was collected by centrifugation at 100 x g for 3 min. The amount of NO released from islets in the media was measured by determining nitrite concentrations following the manufacturer’s instructions (Nitric Oxide Assay Kit, Thermo Fisher).
2. Islet Functionality: Glucose-stimulated insulin secretion (GSIS) was performed using 30 islets per test. Here, islets were transferred into 0.6mL Eppendorf tubes and incubated at 37̊C and 5% CO_2_ in Krebs buffer with bovine serum albumin (0.05% w/v) containing 2.8 mM (D)-glucose for 1 h. After each hour, the buffer was replaced with Krebs buffer containing 2.8 mM glucose (low glucose), followed by Krebs buffer containing 28 mM glucose (high glucose). Islets and the buffer solution were separated by centrifugation (100 x g for 3 min), and the supernatant was removed and subjected to insulin quantification using a rat ELISA immunoassay (Mercodia, USA). The stimulation index for each experimental group was calculated by dividing the insulin concentration in mouse islets exposed to high glucose levels (28 mM glucose) by the one produced in a low glucose environment (2.8 mM glucose).

### Functional enrichment and network analysis of AD-MSC secretome

GEO data was retrieved from our previously published data at GSE199826 [26]. The Reactome Functional Interaction (FI) Network Analysis was performed using ReactomeFIVIz in Cytoscape on highly abundant genes derived from adult adipose tissue-derived mesenchymal stem cells (AD-MSCs). The sample set consisted of AD-MSC mRNA cultured under basal conditions from three donors (18002, 18004, 19002), with three biological replicates for each donor. The gene expression data were filtered to retain highly expressed genes using the following criteria (FPKM > 5 in the 3 replicates), resulting in 6,942 genes. The normalized and filtered gene expression data (log2FPKMs) were used as input for the MCL (Markov Cluster Algorithm) clustering algorithm. We first calculated correlations among genes involved in the same FIs and then used these calculated correlations as weights for the edges (i.e., FIs) in the entire FI network, applied the MCL graph clustering algorithm to the weighted FI network, and generated sub-networks or modules based on the average correlation among the nodes. The MCL network clustering was performed with the following parameters: use absolute values as weights for edges and an inflation parameter of 5. Enriched pathways were identified for each network module using STRING based on KEGG and Reactome pathway annotations. Databases and co-expression were used as active interaction sources to visualize the STRING network edges.

### Statistical analysis

The GraphPad Prism software (version 10.01.02) was used for statistical analysis. Each experiment adhered meticulously to predefined protocols, and results were presented as the mean ± standard deviation (SD). Group differences were assessed through ordinary one-way ANOVA (Analysis of Variance), with post-hoc Tukey tests employed for pairwise comparisons among the groups. A mixed-effect model was also applied to experiments with a repeated measures design. Statistical significance was considered when *p* ≤ 0.05 (adjusted p-value ≤ 0.05 in multiple comparisons). All statistical results are available in Supplementary Excel file 1.

## Results

### In vivo and in vitro models of islet injury

**Fig. 1:**
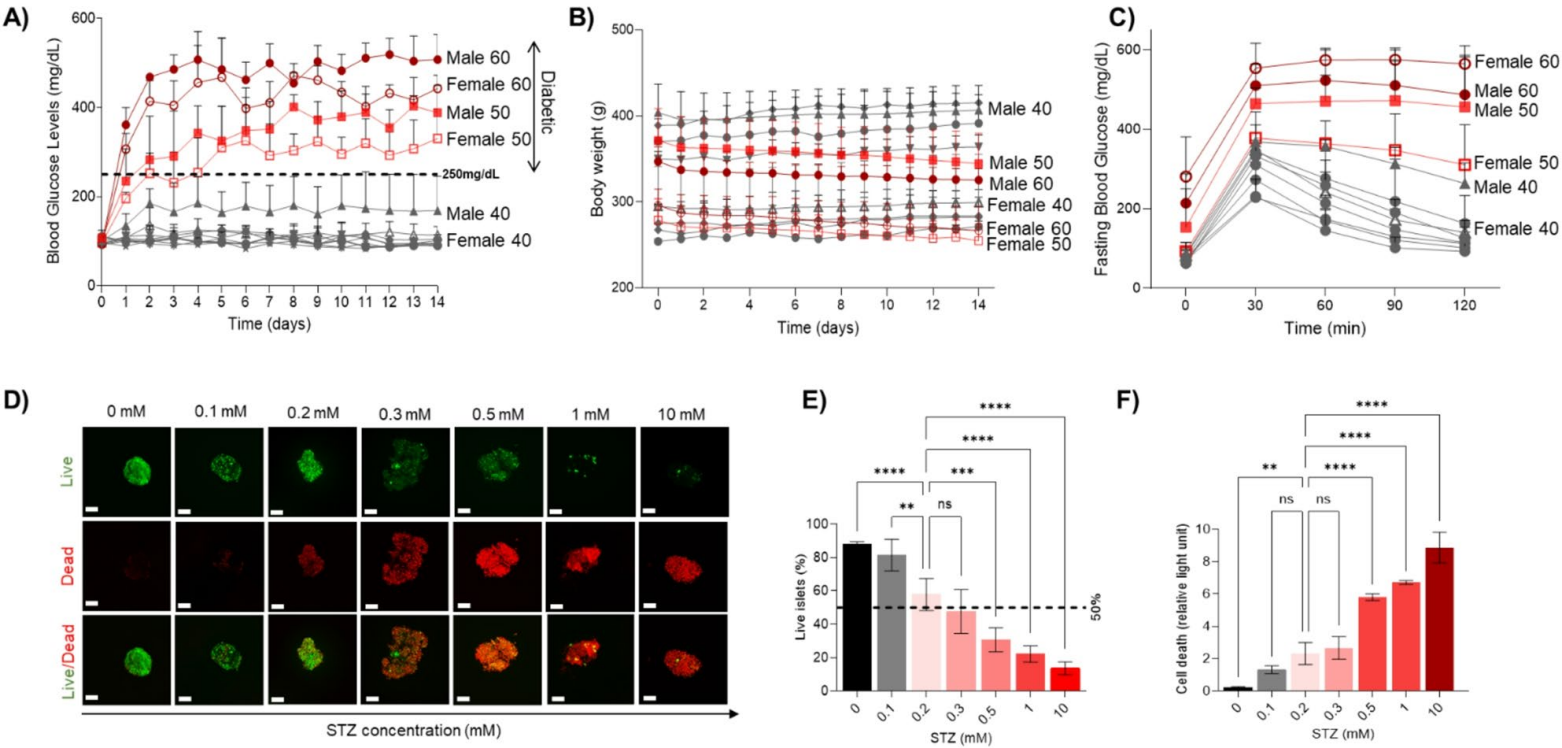
In vivo and in vitro models to study islet injury and impaired islet function.** A** Non-fasting BG levels were measured in rats for 14 days. 60 and 50 mg/kg of STZ resulted in rats developing diabetes. 60 mg/kg resulted in an acute rise in BG levels within less than 24 h. **B** Body weight monitoring showed no significant difference between groups with a low dose, whereas rats receiving 60 and 50 mg/kg STZ either lost weight or were unable to gain weight. **C** IPGTT assessments showed that rats receiving 60 and 50 mg/kg of STZ lost their capacity to regulate glucose levels in response to insulin challenges. **D** Representative live/dead confocal images of islets in the absence and presence of STZ, showing dose escalation decreased cellular viability. Red: dead cells stained with PI, and Green: live cells stained with FDA (scale bar = 75 μm). **E** Live/dead assay shows that STZ significantly reduces islet viability in doses > 0.3 mM, which was **F** further confirmed by a cell death assay.** A**–** C** Statistical significance was determined by a mixed-effect model with a repeated measures design.** E**,** F** Statistical significance was determined by ordinary one-way ANOVA (Analysis of Variance), with post-hoc Tukey tests employed for pairwise comparisons among the groups. ns indicates no significance difference, *indicates *p* ≤ 0.05, **indicates *p* ≤ 0.01, ***indicates *p* ≤ 0.001, and ****indicates *p* ≤ 0.0001. Details of the statistical analysis are reported in Supplementary Excel file 1

To establish an optimized diabetic rat model that slowly developed hyperglycemia with a window for potential intervention, a dose-response study using 10, 20, 30, 40, 50, and 60 mg/kg STZ was performed. At 10, 20, and 30 mg/kg doses, we did not observe any development of diabetes, as reflected by male and female animals having normal BG levels (Fig. 1A). An elevated BG level (average: ∼ 165 mg/dL) at 40 mg/kg was observed only in male rats, but these animals were still not classified as diabetic (> 250 mg/dL). At 50 mg/kg, the BG levels in both male and female rats rose gradually, with male rats becoming diabetic (≥ 250 mg/dL) sooner than females. At 60 mg/kg, all animals demonstrated a faster increase in BG levels, which were sustained for the duration of the experimental protocol (day 14). Rats were more affected at higher STZ doses (40–60 mg/kg). Although male rats were heavier than female rats on day 0, all rats that experienced an increase in BG levels also showed a decrease in body weight, with this weight loss being more pronounced in males. (Figs. 1B). In addition to baseline homeostatic glycemic control, rats that received higher STZ doses had an impaired ability to respond to glucose challenges, as assessed by IPGTT on day 14 (Fig. 1C). Ex vivo histological analysis of the pancreas collected on day 14 demonstrated an increase in islet damage and loss of islet morphology in animals that received 50–60 mg/kg of STZ (Supplementary Fig. 1A-C).

A dose-response study using 0, 0.1, 0.2, 0.3, 0.5, 1, and 10 mM STZ was performed to study the direct effect of STZ on islets. At 0.1 mM, cell damage is insufficient, thereby not allowing for the study of any therapeutic intervention. At 0.2 mM and 0.3 mM, almost 50–60% of pancreatic islets were still alive, with cell death increasing significantly at higher concentrations (≥ 0.5 mM), where total islet death was observed (Fig. 1D-F). Hence, given that 0.2mM STZ caused islet injury but not complete death, this concentration was used to study the protective effects of AD-MSCs. The IC50 (LD50) was determined to be between 0.2 and 0.3 mM STZ by nonlinear regression analysis (Supplementary Fig. 2); however, due to the higher cell death and greater variation (SD) at the 0.3 mM dose, we used the 0.2 mM dose for our in vitro studies.

### The in vivo effect of AD-MSCs in protecting injured islets and maintaining glycemic control

**Fig. 2:**
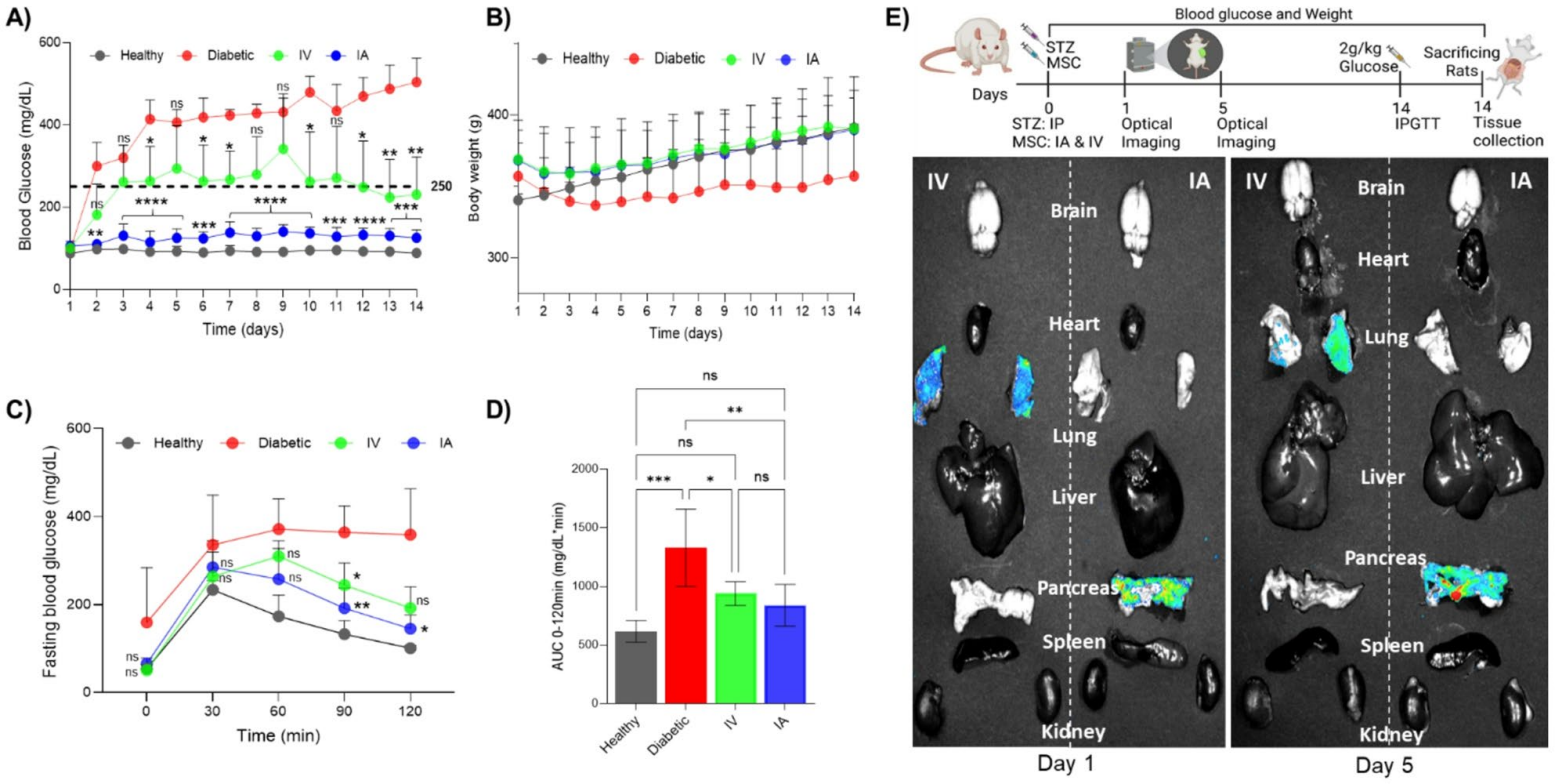
AD-MSC therapeutic effect on diabetic pancreas following IA & IV delivery. **A** 14 days of non-fasting blood glucose monitoring demonstrating that AD-MSCs can protect against STZ-induced diabetes if delivered IA. Although IV AD-MSC delivery was not as therapeutically potent as IA AD-MSC delivery, rats could still regulate their BG levels compared to the untreated diabetic group. **B** Body weight monitoring also shows that, while healthy, IA, and IV AD-MSC groups gained weight, the diabetic group predominantly lost weight (with minimal weight gain). **C** In line with the BG level results, fasting IPGTT on day 14 showed that the diabetic group had lost their capacity to respond to a glucose challenge, and the IV AD-MSC group had delayed glycemic regulation. The IA AD-MSC group could regulate high glucose similarly to the healthy group. **D** The AUC curve (0–120 min) also confirmed this observation. **E** AD-MSC biodistribution on days 1 and 5 post-injection showed pancreas-specific homing of AD-MSCs following IA delivery. **A**–**C** Statistical significance was determined by a mixed-effect model with a repeated measures design. Significant markers reported in the figures A and C show the difference of mice treated IA and IV AD-MSCs compared to diabetic mice. ns indicates no significance difference, *indicates *p* ≤ 0.05, **indicates *p* ≤ 0.01, ***indicates *p* ≤ 0.001, and ****indicates *p* ≤ 0.0001. **D** Statistical significance was determined by ordinary one-way ANOVA (Analysis of Variance), with post-hoc Tukey tests employed for pairwise comparisons among the groups. Details of the statistical analysis are reported in Supplementary Excel file 1

From the STZ dose-titration experiments in rats, the optimal dose to induce a gradual onset of diabetes was 50 mg/kg STZ. Male rats that received no AD-MSCs (diabetic) were unable to regulate their BG levels from day 2 and showed a steady increase in BG levels for 14 days (> 400 mg/dL) (Fig. 2A). When rats received IV AD-MSCs on day 1, although the hyperglycemia was not as pronounced as in the diabetic group, they struggled to regulate their BG levels and were classified as diabetic (≥ 250 mg/dL) from day 3 onward. However, when rats received IA AD-MSCs, glycemic regulation was maintained, with the average BG level remaining between ~ 105 to 140 mg/dL over 14 days (an average of 37% higher than the healthy group, which was between ~ 88 to 98 mg/dL in the healthy group). Over the experimental protocol, rats treated with AD-MSCs showed an increase in body weight, independent of whether the cells were given IV or IA (Fig. 2B). Dynamic glucose regulation was preserved in healthy rats and those receiving IA AD-MSCs, followed by those receiving IV AD-MSCs (Fig. 2C, D). The greater protective effect of IA AD-MSCs was likely due to their direct delivery to the pancreas. Our biodistribution data show only labeled AD-MSCs in the pancreas with IA injection on days 1 and 5. In the IV group, no signal was detected in the pancreas; however, a significant signal was observed within the lungs due to the pulmonary first-pass effect (Fig. 2E).

### The in vitro effect of AD-MSCs in protecting injured islets and maintaining islet viability

**Fig. 3:**
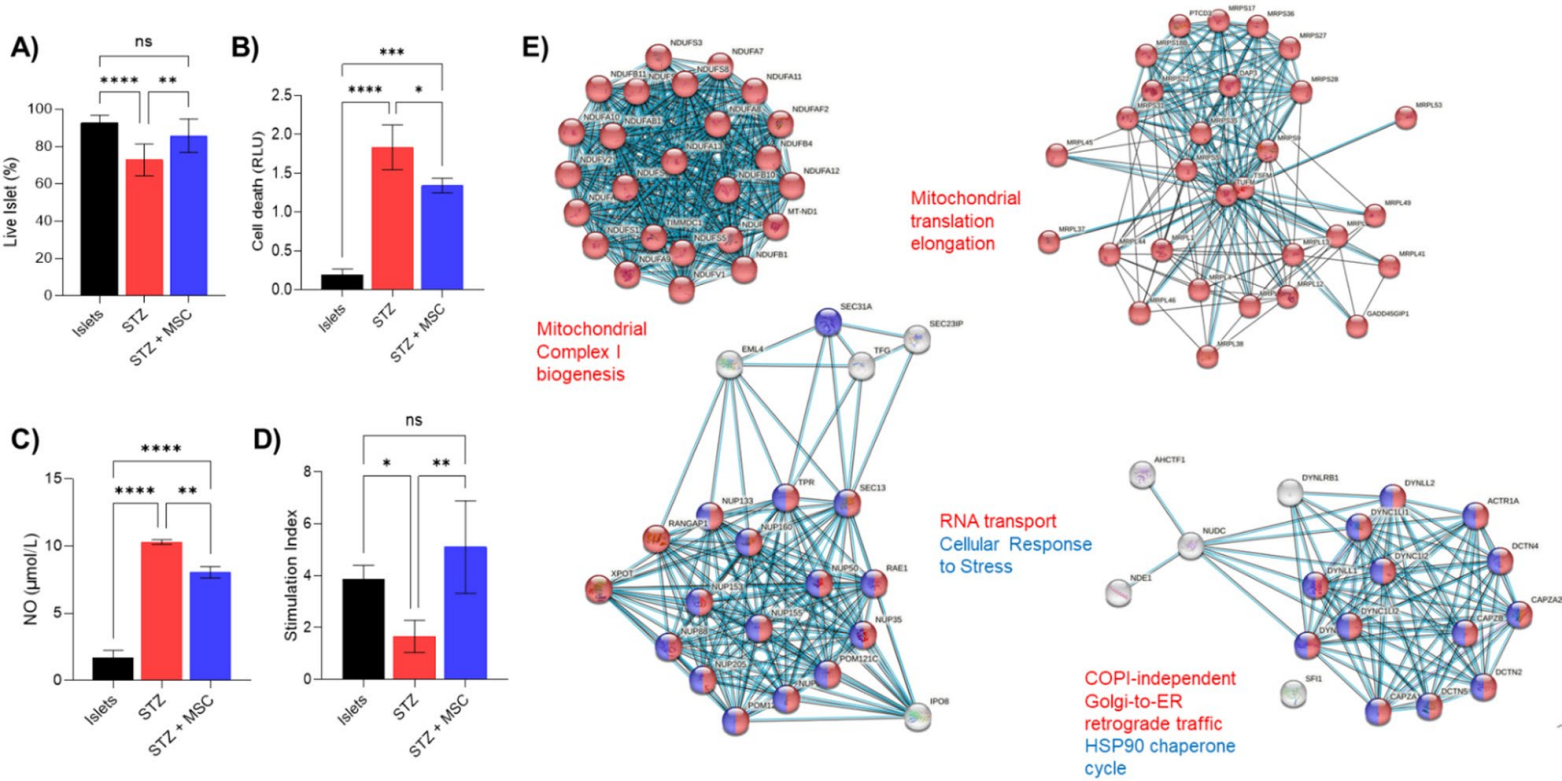
AD-MSCs protect the stressed rat islets and support their function.** A** Live/Dead assay showing that AD-MSCs can significantly increase the viability of STZ-stressed islets, also confirmed by **B** cell death assays. **C** NO measurements show that NO production is reduced in the presence of AD-MSCs (unregulated NO production causes cell death; thus, NO reduction has a protective effect). **D** The insulin release functionality of rat islets, presented as the stimulation index, shows improvement in the presence of AD-MSCs. In the absence of AD-MSCs, this activity is significantly lower. **E** Functional annotation and network analysis of the AD-MSC secretome identified several protective and housekeeping processes, including mitochondrial complex biogenesis, RNA transport, and ER-related traffic, as top-enriched pathways for the top 4 modules (sub-networks). Statistical significance was determined by ordinary one-way ANOVA (Analysis of Variance), with post-hoc Tukey tests employed for pairwise comparisons among the groups. ns indicates no significance difference, *indicates *p* ≤ 0.05, **indicates *p* ≤ 0.01, ***indicates *p* ≤ 0.001, and ****indicates *p* ≤ 0.0001. Details of the statistical analysis are reported in Supplementary Excel file 1

Islets exposed to 0.2 mM STZ for 48 h, both in the presence and absence of AD-MSCs, exhibited a loss of viability and an increase in cell death. This was, however, mitigated with AD-MSCs, with Live/Dead assays showing stressed islets demonstrating significantly lower viability when AD-MSCs are not present compared with islets co-cultured with AD-MSCs (68.4 ± 3.1 and 80.2 ± 2.8%, respectively; *p* < 0.01) (Fig. 3A and Supplementary Fig. 3A). The cell death assay also showed ∼1.5-fold higher cell death in the absence of AD-MSCs (*p* < 0.05) (Fig. 3B). As a mediator of cell death, NO measurement also showed a significant decrease in the presence of AD-MSCs (Fig. 3C). Furthermore, in the absence of AD-MSCs, there is a reduction in the insulin release from stressed islets, as shown by their stimulation index (AD-MSC: 5.1 ± 1.8 vs. STZ: 1.6 ± 0.6; *p* < 0.001) (Fig. 3D). In response to STZ, there seems to be a combination of insulin secretion and insulin leak/release (uncontrolled insulin release), most probably due to the islet damage.

MSCs mainly exert their effects through paracrine activity; thus, we have examined the AD-MSCs’ secretome [26]. The Reactome pathways-based FI network clustering analysis was performed by converting a non-weighted FI network to a weighted network using correlations among genes. Only modules with more than 20 genes and an average correlation >0.5 were selected and used in the FI sub-network building. The top 4 FI sub-networks (modules) were enriched in mitochondrial complex biogenesis, mitochondrial translation elongation, cellular response to stress, RNA transport, and COPI-independent Golgi-to-ER retrograde trafficking (Fig. 3E). In addition to the modules emphasized below, the Reactome-based enrichment also included categories related to ribosome, mRNA processing/splicing, and endocytosis; full rankings and statistics are provided in Supplementary Excel File 2, and all detected modules are visualized in Supplementary Fig. 3B.

## Discussion

Our study aimed to evaluate the therapeutic efficacy of AD-MSCs in preventing diabetes in the context of progressive β cell loss. Our data shows that STZ induces islet injury and impairs islet functionality through NO-mediated cell death. In vivo, this results in the development of diabetes in rats, with the onset and severity directly influenced by the STZ dose. While AD-MSCs were shown to protect and restore islet functionality, this effect was predominantly seen when AD-MSCs were delivered directly into the pancreas by IA injection. In contrast, IV injections provided minimal benefit, with a markedly reduced therapeutic response compared to precision delivery of AD-MSCs into the pancreas.

The use of STZ, with a short half-life, to create diabetic models is based on its ability to damage pancreatic islets (specifically β cells), thereby causing an insulin secretion shortage and increased BG levels [32]. In our study, a 50 mg/kg STZ dose in rats provided a therapeutic window for evaluating interventions targeting the prediabetic pancreas. To ensure the treatment opportunity window was not missed, the treatment was initiated early, as the β cell damage increased exponentially from day one. Although diabetes induction was largely consistent across rats (without AD-MSC therapy), some still did not respond to the STZ challenge accordingly and were excluded. While these are inbred rats, this could be due to individual differences between animals, including the slight age difference that may affect their response to STZ, resulting from metabolic differences or variations in administration. Our data also showed a sex difference, with males being more responsive to STZ-induced damage; the level of response heterogeneity to the STZ was also lower in males than in females, confirming findings from other studies [33–37].

Our results show that both IA and IV administration of AD-MSCs mitigated diabetes onset and helped regulate glycemic levels compared to the untreated diabetic group. However, rats receiving AD-MSCs through IA injection maintained basal and dynamic glucose regulation far better than the IV AD-MSC and diabetic group. This is likely due to the precise pancreatic localization of AD-MSCs following IA delivery, as opposed to off-target accumulation in filtering organs (i.e., the lungs) observed with IV delivery. MSC homing to organs other than the pancreas [38–40] results in limited and non-sustainable therapeutic effects. AD-MSC administration through the IV route most likely functions through systemic immunomodulation and anti-inflammatory activities, with less dependence on direct islet interaction [41, 42]. In vitro experiments corroborated these findings, showing that AD-MSCs can protect stressed islets through improved viability, reduced cell death, and NO production (a critical mediator of β cell death [43–47]) resulting in maintenance of insulin secretion activity. Increased viability could be due to decreased NO production [48] (decreased cell death) or increased cell proliferation. Moreover, insulin secretory activity was maintained and increased in islets co-cultured with AD-MSC, confirming a previous report [49]. Given that these experiments were performed in a trans-well system, the protective effects are likely due to the paracrine effects of AD-MSCs, which may partially explain the effects seen with IV administration in vivo. The secretome analysis detected several housekeeping, bioenergetic, and protective processes, further supporting the paracrine therapeutic role of AD-MSCs [50]. The Reactome FI network clustering analysis highlighted enrichment of pathways related to mitochondrial complex biogenesis, mitochondrial translation elongation, cellular response to stress, RNA transport, and COPI-independent Golgi-to-ER retrograde trafficking. These results suggest that MSCs may support islet survival by enhancing mitochondrial function, maintaining cellular protein homeostasis, and modulating stress-response pathways. In particular, the mitochondrial pathways indicate a role for MSCs in maintaining energy metabolism and mitigating oxidative damage, while RNA transport and retrograde trafficking pathways indicate potential regulation of protein synthesis and intracellular transport, which are crucial for islet function. While our current study provides associative evidence linking these processes to MSC-mediated protection, we did not experimentally validate these mechanisms in vitro or in vivo, which we acknowledge as a limitation. Future studies incorporating targeted perturbations of these pathways, coupled with functional assays of islet viability and insulin secretion, will be essential to confirm the mechanistic links between MSC action and the pathway-level insights revealed by our analysis.

Promising results from AD-MSC’s pre-clinical testing have prompted several clinical trials [6, 51], mainly for local applications. We selected AD-MSCs for this prevention-focused study based on practical and translational considerations, including high cell yield from minimally invasive liposuction procedures [52]. Moreover, well-established clinical workflows for adipose procurement and processing, along with the feasibility of autologous sourcing without ethical constraints for AD-MSCs, all support scalable manufacture and timely delivery [53]. In contrast, bone marrow (BM)-MSCs require more invasive harvesting with lower typical yields, and umbilical cord (UC)-MSCs are allogeneic, and depend on the availability of qualified donor tissue banking and release criteria. In addition, therapeutic profiles of MSCs can vary by tissue source and donor; accordingly, proliferation, secretome composition, and immunomodulation heterogeneity is increasingly recognized, and this reinforces the need to match the MSC source to target mechanism. Given these considerations, AD-MSCs offered a pragmatic balance of availability, scalability, and paracrine activity for precision IA delivery in our preclinical design, while acknowledging that BM-MSCs or UC-MSCs may be preferred for other indications. The technique used for IA delivery to the pancreas was developed [9, 10] to emulate what can clinically be performed by endovascular delivery using minimally invasive image-guided procedures. Once AD-MSCs reach the injured pancreas, they can come into close contact with damaged islets, enabling them to release therapeutic anti-apoptotic [21–23], regenerative [17, 18], anti-inflammatory, and immunomodulatory factors [19, 20] that modulate the surrounding inflamed microenvironment. Closer contact with target cells also facilitates cell-cell communication, leading to faster healing and enhanced protection. MSCs can also transfer metabolic and bioenergetic cargo to stressed cells [54–56]. Here, direct physical contact will play a crucial role in transferring mitochondria from MSCs to damaged islets, thereby restoring the bioenergetic capacity of β cells [57–59]. Moreover, MSCs can create a tolerogenic niche by direct interaction with immune cells and by secretion of regulatory molecules [60–62]. In line with our observations, it has been demonstrated that AD-MSCs prevent the onset of T1D by immune checkpoint blockade in NOD mice [63] and improve glycemic regulation in STZ-stressed mice [64].

## Conclusion

In conclusion, local pancreatic IA delivery of AD-MSCs effectively prevents the onset of diabetes in a pre-clinical rat model. Comparing the outcomes of the IV and IA administration underscores the importance of delivery for maximizing the therapeutic benefits of AD-MSCs. While this study highlights the protective effects of AD-MSCs, further research is needed to assess their regenerative impact on islets. Future studies will also explore optimal administration timing, extended study durations, and the underlying molecular mechanisms of AD-MSC therapy.

## Supplementary Information

Below is the link to the electronic supplementary material.

Supplementary Material 1.

Supplementary Material 2.

Supplementary Material 3.

## Data Availability

The authors confirm that the data supporting the findings of this study are available within this article and its Supplementary material. Raw data that support the findings of this study are available from the corresponding author upon reasonable request.
