# Supplementary material for: Preventing the onset of diabetes with precision delivery of mesenchymal stem cells to the pancreas in a preclinical model
Source: Stem Cell Res Ther. 2025 Nov 23;16:687. doi: 10.1186/s13287-025-04807-3 (PMC12751359; doi:10.1186/s13287-025-04807-3)
Supplement: Supplementary file 3 — Supplementary Material 3. [file 13287_2025_4807_MOESM3_ESM.docx]

**SUPPORTING INFORMATION
Preventing the onset of diabetes with precision delivery of mesenchymal stem cells to the pancreas in a preclinical model**

Reza Yarani^1,2^†, Rosita Primavera^1^†, Shashank Chetty^1^, Jing Wang^1^, Simranjeet Kaur^2^, Martin Haupt-Jorgensen^3^, Flemming Pociot^2,4^*, Avnesh S Thakor^1^*

^1^ Interventional Radiology Innovation at Stanford (IRIS), Department of Radiology, Stanford University, Palo Alto, CA 94304, USA

^2^ Translational Type 1 Diabetes Research, Department of Clinical Research, Steno Diabetes Center Copenhagen, 2730 Herlev, Denmark

^3^ Bartholin Institute, Department of Pathology, Rigshospitalet, Copenhagen University, 2200 Copenhagen, Denmark

^4^ Institute for Clinical Medicine, Faculty of Health and Medical Sciences, University of Copenhagen, 2200 Copenhagen, Denmark

†Equal contribution

*Corresponding authors:[flemming.pociot@regionh.dk](mailto:flemming.pociot@regionh.dk); [asthakor@stanford.edu](mailto:asthakor@stanford.edu)

**B**

**
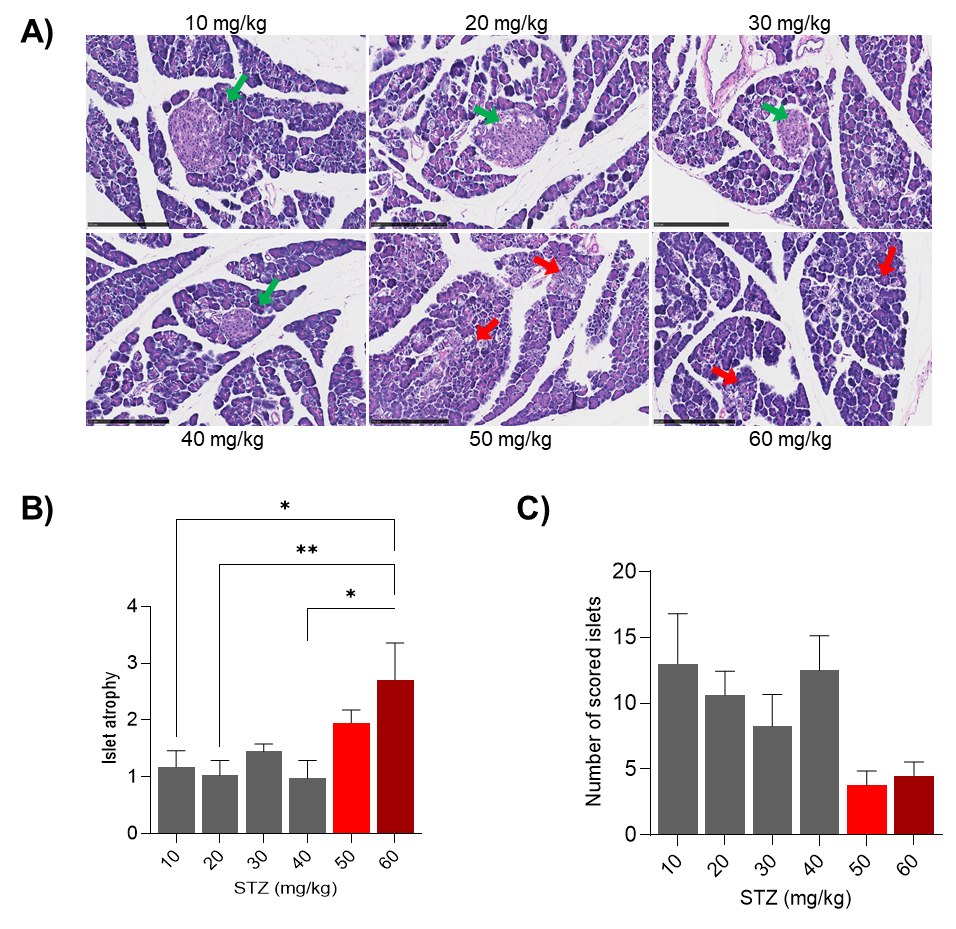
**

**Supplementary Figure 1. A)** H&E staining for islet structure assessment also reveals higher islet damage, as reflected in islet atrophy and vacuolar degeneration, for the 60 and 50 mg/kg groups. Red arrows indicate destroyed islets, and green arrows show healthy islets. **B)** Islet atrophy and **C)** number of scored islets. Islet atrophy was higher for rats receiving 60 and 50 mg/kg of STZ. Histological analyses were conducted by assigning a score based on the degree of damage observed in each group within the tissue under examination: 0 = no damage; 1 = minimal (1–10%); 2 = mild (10%−35%); 3 = moderate (36%−50%); and 4 = severe (>50%). Specifically, the percentage of damage refers to the extent of damage within the islets and was assessed by evaluating islet atrophy (as shown in Figure C) and vacuolar degeneration (not shown, as it received the same scoring as atrophy). These scoring criteria were adapted from Oyouni et al. A minimum of N=4 samples from each group were included from each paraffin block, and two non-overlapping sections were analyzed at five distinct positions. We minimized potential bias through several key measures: (i) slides were evaluated by a researcher with expertise in pancreatic pathology, and a pathologist, both of whom were blinded; (ii) the order of the slides was randomized prior to scoring; (iii) we established objective criteria with visual exemplars for each scoring category. Additionally, scoring was performed on a subset of slides to assess reproducibility, and the intra-rater agreement exceeded our preset threshold.


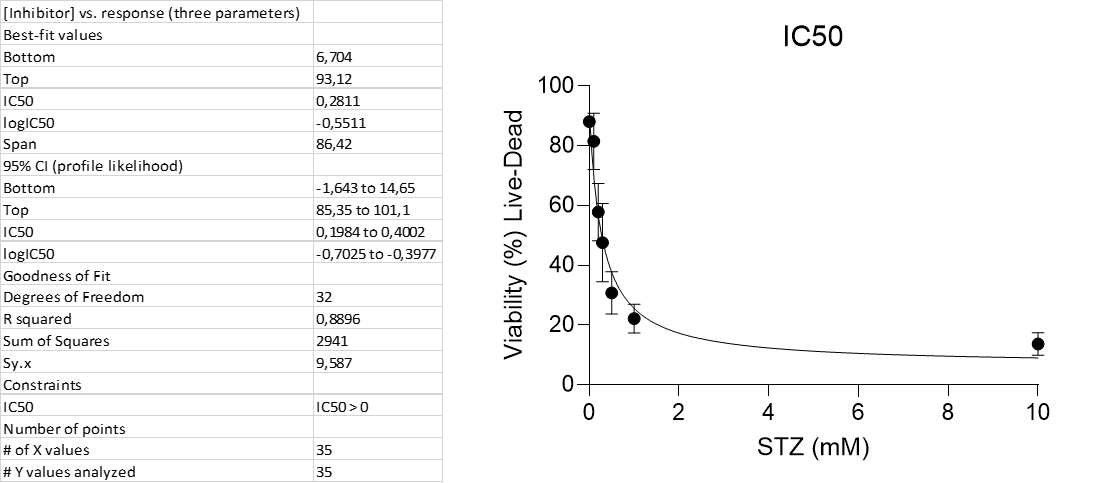


**Supplementary Figure 2.** IC50 (LD50): Nonlinear regression analysis using GraphPad defines IC50 as 0.2811 mM. For the STZ dose–response, pancreatic islets were exposed to 0–10 mM STZ for 48 hrs, and cell death was measured. The IC50 or LD50 (half‑maximal lethal concentration) is reported as the STZ concentration that reduces viability to 50% of the untreated control at 48 hrs. The fitted curve placed the LD50 between 0.2 and 0.3 mM, consistent with the observed inflection; because 0.3 mM produced greater variance and excessive death, 0.2 mM was selected for subsequent mechanistic assays.


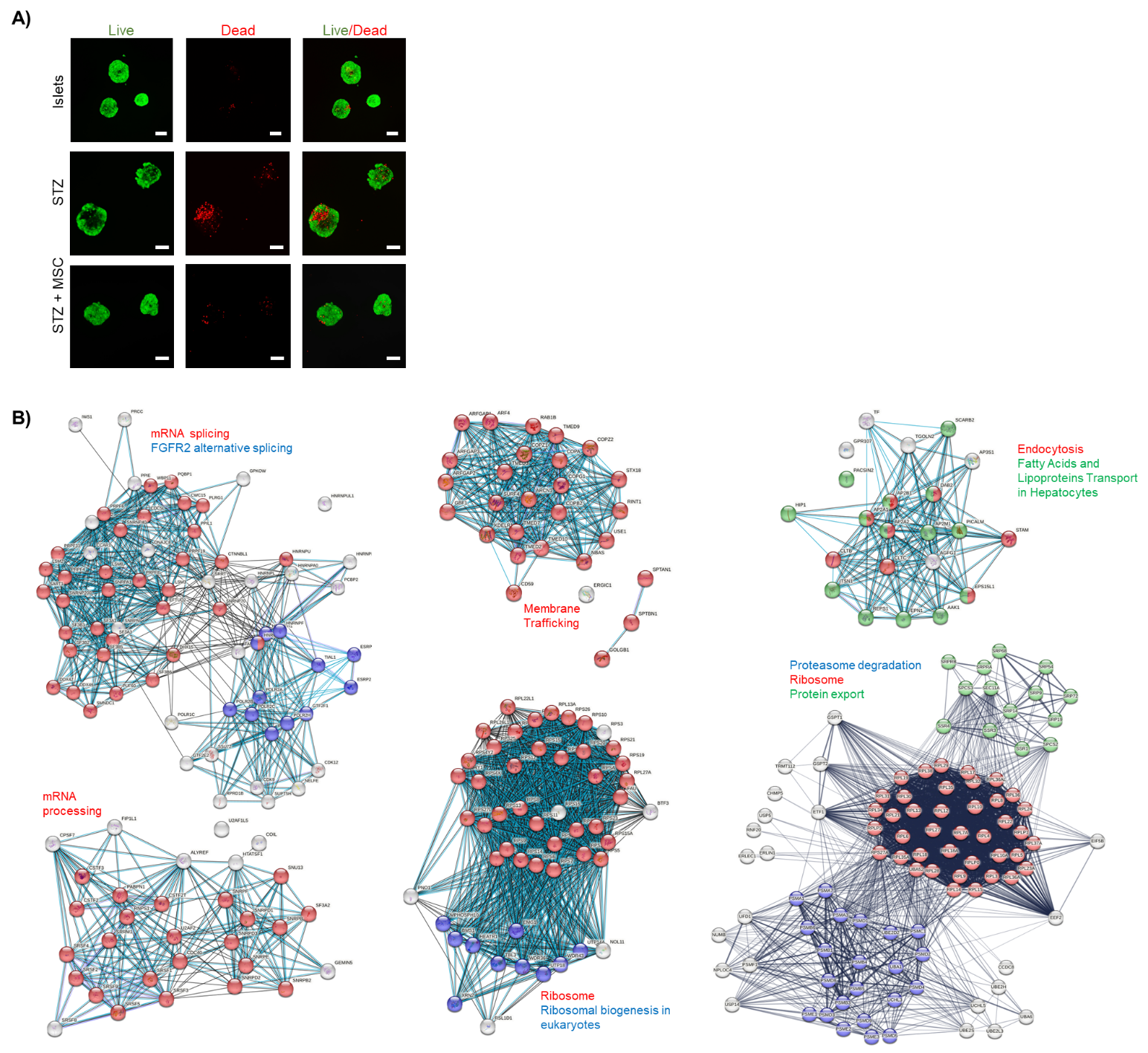


**Supplementary Figure 3**. **A)** Representative Live/Dead images of rat islets co-cultured without and with AD-MSCs in presence or absence of STZ. Red is the dead cells stained with PI, and green is the live cells stained with FDA (scale bar = 100 µm). **B)** Functional annotation and network analysis of the AD-MSC secretome (sub-networks).

**References:**

1 Oyouni, A. A. A. *et al.* Melatonin ameliorates the adrenal and pancreatic alterations in streptozotocin-induced diabetic rats: Clinical, biochemical, and descriptive histopathological studies. *Front Vet Sci* **9**, 1016312 (2022). <https://doi.org/10.3389/fvets.2022.1016312>
